# Hormonal content and potency of oral contraceptives and breast cancer risk among young women

**DOI:** 10.1038/sj.bjc.6600691

**Published:** 2003-01-28

**Authors:** M D Althuis, D R Brogan, R J Coates, J R Daling, M D Gammon, K E Malone, J B Schoenberg, L A Brinton

**Affiliations:** 1 National Cancer Institute, Rockville, MD, USA; 2 Rollins School of Public Health, Atlanta, GA, USA; 3 Centers for Disease Control and Prevention, Atlanta, GA, USA; 4 Fred Hutchinson Cancer Research Center, Seattle, WA, USA; 5 University of North Carolina, Chapel Hill, NC, USA; 6 New Jersey State Department of Health and Senior Services, Trenton, NJ, USA

**Keywords:** breast cancer, oestrogen, formulation, oral contraceptives, progestin

## Abstract

Recent use of oral contraceptive pills is associated with a modest risk of breast cancer among very young women. In this US population-based case–control study, we evaluated whether the excess risk associated with recent oral contraceptive use is ubiquitous for all pill types or attributable to specific oral contraceptive preparations. Hormonal content and potency of combination oral contraceptives used for the longest duration within 5 years of interview for breast cancer cases aged 20–44 years (*N*=1640) were compared with age-matched community controls (*N*=1492). Women who recently used oral contraceptives containing more than 35 *μ*g of ethinyl oestradiol per pill were at higher risk of breast cancer than users of lower dose preparations when compared to never users (respective relative risks of 1.99 and 1.27, *P*_trend_<0.01). This relationship was more marked among women <35 years of age, where risks associated with high- and low-dose ethinyl oestradiol use were 3.62 and 1.91 (*P*_trend_<0.01), respectively. We also found significant trends of increasing breast cancer risk for pills with higher progestin and oestrogen potencies (*P*_trend_<0.05), which were most pronounced among women aged <35 years of age (*P*_trend_<0.01). Risk was similar across recently used progestin types. Our findings suggest that newer low-potency/low oestrogen dose oral contraceptives may impart a lower risk of breast cancer than that associated with earlier high-potency/high-dose preparations.

A small study of women with early-onset breast cancer published in 1983 initially sparked the debate about combination oral contraceptives and breast cancer by suggesting that a woman's risk of breast cancer increased if she used oral contraceptives early in life, particularly pills with high progestin potency (Pike *et al*, 1983). Evidence from a multitude of case–control and cohort studies conducted in the 1980s and early 1990s subsequently found a modest (approximately 20–40%) but consistent excess in breast cancer risk associated with recent oral contraceptive use among women younger than 45 years of age (Collaborative Group on Hormonal Factors in Breast Cancer, 1996a). Whether this excess risk is ubiquitous for all pill types or attributable to specific oral contraceptive preparations is considerably less well studied.

The majority of investigations that report risk associated with specific oral contraceptive preparations among women younger than 45 years of age assess lifetime exposure to oral contraceptives instead of the more relevant exposure, recent use (Pike *et al*, 1983; Vessey *et al*, 1983,^1989^; McPherson *et al*, 1987; Jick *et al*, 1989; UK National Case–Control Study Group, 1989; White *et al*, 1994; Brinton *et al*, 1995; Ursin *et al*, 1998). Consequently, there have been mixed reports of increased breast cancer risk associated with high progestin potency pills (White *et al*, 1994; Pike *et al*, 1983), and oestrogen content, specifically ethinyl oestradiol use (McPherson *et al*, 1987; Jick *et al*, 1989) and long-term use of high-dose oestrogen pills (≥50 *μ*g) (UK National Case–Control Study Group, 1989; Ursin *et al*, 1998). Three of the nine studies examining hormonal content of combination oral contraceptives among women younger than 45 years reported no association with either oestrogen or progestin content (Vessey *et al*, 1983,^1989^); Brinton *et al*, 1995), but two of three employed unadjusted estimates of risk associated with ever use and the third, an early analysis of the data presented in this paper, focused on duration of use. Although a collaborative reanalysis assessing 26 studies with data on hormonal content of combined oral contraceptive preparations (which included data from this study) observed no association of first used, longest used, or most recently used oral contraceptive formulation with breast cancer risk, these results were based on a large proportion of older women for whom oral contraceptive use is not a breast cancer risk factor (Marchbanks *et al*, 2002): 66% of data analysed were from women 45 years of age or older and subgroup analyses among younger women were limited (Collaborative Group on Hormonal Factors in Breast Cancer, 1996b).

Since Food and Drug Administration (FDA) approval in 1960, the oral contraceptive pill has been formulated using two oestrogens and nine different progestins, varying in both dose and potency (Piper and Kennedy, 1987). Oral contraceptives underwent considerable changes in formulation during the 1970s and 1980s, so that by the 1990s they contained approximately one-fourth the oestrogen and one-tenth the progestin of earlier versions (Boonstra *et al*, 2000). Thus, it is plausible that risk associated with oral contraceptive use may be altered over time because of changes in formulation. In fact, recent investigations of the association of oral contraceptives with ovarian cancer, one of which was from a large population-based study (Schildkraut *et al*, 2002), suggest that formulations with higher progestin (Schildkraut *et al*, 2002) or oestrogen potency (Rosenberg *et al*, 1994; Rosenblatt *et al*, 1992) conferred a greater reduction in risk of ovarian cancer than those with lower potency. In this study, we investigate whether differences exist in breast cancer risk associated with recent oral contraceptive use for newer low-potency pills and older higher-dose preparations. This paper reports a comprehensive analysis of the relationship of oral contraceptive formulation with breast cancer risk by examining oestrogen and progestin type, dose, and potency using data from a large population-based case–control study specifically designed to answer this question.

## Materials and methods

### Subjects

Brinton *et al* (1995) previously described this population-based case–control study. Briefly, the breast cancer case subjects in this investigation included patients, 20–54 years of age, newly diagnosed with *in situ* or invasive breast cancer in 1990–1992 while living in either the metropolitan areas of Atlanta, Georgia or Seattle/Puget Sound, Washington, or five counties of central New Jersey. Regional and governmental institutional review boards approved the study protocol. Hospital records of eligible patients were abstracted to document details on the clinical and pathologic characteristics of the breast cancers. Control subjects in the three geographic areas were ascertained through random-digit dialling, with a 90.5% screening response rate.

### Data collection

Following written informed consent, participants were interviewed in person about oral contraceptive pill use from menarche until the date of interview. Pregnancies and other life events were first marked on a month-by-month family planning calendar to serve as a frame of reference and to aid recalling dates of changes in contraceptive use over time. Months of oral contraceptive use were subsequently shaded on the calendar. On a complementary worksheet, interviewers recorded the start and stop dates for each episode of pill usage, the brand name, reason for use, and reason for discontinuing use. Colour photographs of oral contraceptives and listings of the year they were first marketed in the United States were shown to help participants identify the brand name of each oral contraceptive used.

In addition, in-person interviews included questions about demographic factors, reproductive and menstrual history, use of exogenous hormones, medical and screening history, anthropometry and physical activity, adolescent diet, alcohol consumption, smoking, occupation, family history of cancer, and certain lifestyle factors. Interviews were obtained from 2002 eligible patients (86%) and 2009 eligible control subjects (78%). A comparison of respondents to nonrespondents who were willing to complete an abbreviated questionnaire reported no important differences for the distributions of variables assessed in this study (Madigan *et al*, 2000).

### Exclusions

This investigation included only women younger than 45 years of age (*N*=3132), the age group for which oral contraceptive use is a risk factor for breast cancer (Collaborative Group on Hormonal Factors in Breast Cancer, 1996a). We excluded from analysis patients who indicated on interview that they either did not have a residential telephone (*n*=28) or that they had a previous diagnosis of breast cancer (*n*=19). We also excluded 16 of the young women who used solely progestin-only pills. After the exclusions, 1640 cases and 1429 controls remained in this investigation.

### Defining oral contraceptive formulation and potency

Oral contraceptive use was defined as use of combination oral contraceptive pills for a total of 6 or more months. Women who never used oral contraceptives or who used them for less than 6 months were classified as nonusers. For each oral contraceptive preparation used, we obtained information on oestrogen and progestin content and dose from the Physician's Desk Reference available during the time that the preparation was marketed in the United States (Table 1

**Table 1:** Combination oral contraceptive use among premenopausal WISH participants, by brand name and crossclassified by pill content and potency

	**Low progestin/high oestrogen potency**		**Low progestin/low oestrogen potency**		**High progestin/low oestrogen potency**	**Low progestin/high oestrogen potency**
**Progestin **	**Mestranol**	**Ethinyl oestradiol**	**Ethinyl oestradiol**	**Mestranol**	**Mestranol**	**Ethinyl oestradiol**
Chlormadinone acetate						
Dimethisterone						
Ethynodiol diacetate			Demulen 1/35^a^ (1/35)	Ovulen^a^ (1/100)	Demulen 1/35^a^ (1/35)	Ovulen^a^ (1/100)
			Demulen 1/50^a^ (1/50)		Demulen 1/50^a^ (1/50)	
Medroxyprogesterone acetate			Provest^a^ (10/50)		Provest^a^ (10/50)	
Norethynodrel				Enovid 10 mg^a^ (9.85/150)		Enovid 10 mg^a^ (9.85/150)
Norethindrone	*Genora 1/50*^a^ (1/50)	Brevicon (0.5/35)		Ortho-Novum 10 mg^a^		Ortho-Novum 10 mg^a^
	Norinyl 1/50 (1/50)	Modicon (0.5/35)		(10/60)		(10/60)
	Ortho-Novum 1/50	Nelova 1/35e^a^ (1/35)		*Norinyl 10 mg*^*a*^ *(10/60)*		*Norinyl 10 mg*^a^ *(10/60)*
	(1/50)	Norinyl 1/35 (1/35)				
		Tri-norinyl (0.5 : 1 : 0.5/35)				
		*ORF 1557-BE*^*a*^ *(0.5/35)*				
		*ORF 1557-BF*^*a*^ *(1/35)*				
		Ortho-novum 1/35 (1/35)				
		Ortho-novum 10/11 (0.5 : 1/35)				
		Ortho-novum 7/7/7 (0.5 : 0.75 : 1/35)				
		Ovcon 35 (0.4/35)				
Norethindrone acetate		Loestrin 1.5/30 (1.5/30)		Norlestrin 1/50^a^ (1/50)	Ovral (0.5/50)	
		Loestrin 1/20 (1/20)		*Norlestrin 2.5/50*^*a*^ *(2.5/50)*		
		*Norlestrin var red*^*a*^ *(1/20)*		*Norlestrin low*^*a*^ *(0.5/50)*		
		Zorane 1.5/30^a^ (1.5/30)		*Norlestrin var black*^a^		
		Zorane 1/20^a^ (1/20)		(2/40)		
						
Norgestrel		Lo/Ovral (0.3/30)	Ovral (0.5/50)			
		*Ovral blue*^*a*^ *(0.15/30)*				
		*Ovral brown*^a^ *(0.15/15)*				
Levonorgestrel		*Levlen (0.15/35)*				
		Nordette (0.15/30)				
		Tri-levlen (0.05 : 0.075 : 0.125/30 : 40 : 30)				
		Triphasil (0.05 : 0.075 : 0.125/30 :40 : 30)				

a Oral contraceptives no longer marketed in the United States (not listed in the 2002 Physician's Desk Reference).

*Note*: Progestin (mg) and oestrogen dose (*μ*g) is indicated after each oral contraceptive brand name in parentheses. Italicized brand names were added to the potency scheme based on progestin and oestrogen content as evaluated by the authors.

). Oral contraceptive preparations were grouped according to oestrogen content and dose (⩽50 *μ*g, 51–99 *μ*g, or 100+*μ*g mestranol; ⩽35 *μ*g or 36+ *μ*g ethinyl oestradiol) and progestin content (dimethisterone, chlormadinone acetate, norethynodrel, ethinyl diacetate, norethindrone, norethindrone acetate, norgestrel, and levonorgestrel).

In addition to assessing breast cancer risk associated with oestrogen and progestin types, we also evaluated oestrogen and progestin potencies for each pill preparation. To this end, we classified combined oral contraceptive pills into hormone potency categories using a method described in a standard pharmacy reference text (*Drug Facts and Comparisons*, 1997), which has been used previously by the FDA (Piper and Kennedy, 1987). Oestrogen potency data were based on mouse uterine weight gain and human uterine volume analysis and progestin potency on delay of menses data in humans and the degree of glycogen incorporation in human endometrial vacuoles. The categorisation scheme simultaneously ranks oestrogen and progestin potencies as low, intermediate, or high for each product. For this analysis, we condensed this classification scheme to create a categorical variable with four levels describing hormone potency: high progestin/high oestrogen, high progestin/low oestrogen, low progestin/high oestrogen, or low progestin/low oestrogen potency. High- and low-potency preparations as described in the standard pharmacy text were classified as high and low, respectively, for this analysis. We divided the preparations that fell in the intermediate oestrogen potency category (in the pharmacy text) into high- and low-potency groups for this analysis. Oral contraceptive formulations with 35 *μ*g ethinyl oestradiol or 50 *μ*g mestranol were classified as high potency since these doses are approximately biologically equivalent (Brody *et al*, 1989). Those with less than 35 *μ*g ethinyl oestradiol or 50 *μ*g mestranol were classified as low potency. Intermediate progestin potency from the classification scheme was assigned to the low progestin potency group for this analysis. Products not included in the textbook potency classification scheme were evaluated for hormonal content and dose, and assigned to a potency group by the authors (listed in italics in Table 1).

Since oral contraceptive users reported multiple episodes of usage, determining how to assess a series of pill preparations was complex. A reanalysis of data from 54 epidemiologic studies and over 100 000 women found that of all the ways to look at oral contraceptive use, recency was both independent and the strongest predictor of breast cancer risk (Collaborative Group on Hormonal Factors in Breast Cancer, 1996a). In that study, risk was attenuated after discontinuation of oral contraceptives and, 10 years after discontinuation of pills, risk was similar to never-users. Furthermore, the relationship between recency and breast cancer risk was not materially affected after adjusting for duration of use or use prior to childbearing or prior to 25 years of age. This has also been shown in a previous publication of the data from the present investigation (Brinton *et al*, 1995). Thus, in this study, we have assessed content of the oral contraceptive preparations used for the longest duration in the period (1) within 5 years prior to diagnosis and (2) within 10 years prior to diagnosis.

### Statistical methods

We describe the use of combination oral contraceptives among control subjects. Frequency distributions are used to summarise categorical variables and means, standard deviations, and medians for continuous variables.

The relationship between combination oral contraceptive use (ever use and recency of use) and breast cancer risk is reported overall and for two age strata: <35 and 35–44 years of age. Risk associated with hormonal content and potency was also assessed within these age strata after restricting the population to nonusers and those who used oral contraceptives (1) within 5 and (2) within 10 years of diagnosis (cases)/interview (controls). We also evaluated whether risk estimates for recently used pill formulations varied by duration of oral contraceptive use by repeating the above-described analyses separately for women who used pills for less than 5 years or for 5 or more years in total.

Relative risks (RRs) and their 95% confidence intervals (CIs) were used to assess whether breast cancer risk was elevated by oral contraceptive use (ever *vs* never; recency of use) or by specific hormonal preparations, stratifying by age group and controlling for potential confounders. We used unadjusted and adjusted logistic regression models to obtain maximum likelihood estimates of the odds ratio, which were used to approximate RR (Breslow and Day, 1980). To test whether the relationship between ever oral contraceptive use and breast cancer diagnosis was modified by age at diagnosis/interview on a multiplicative scale, both the main effects and their interaction term were evaluated in the model and the statistical significance of the interaction term was assessed. All final models adjusted for study site (Atlanta, Seattle, New Jersey), age (continuous), race (white, black, others), number of mammograms within 5 years of interview (continuous), menopausal status (pre-, postmenopausal), age at menarche (<12, 12, 13, 14+ years), a combination variable including number of full-term births and age at first birth (no birth, 1 birth at age <25 years, 1 birth at age ≥25 years, 2 births at age <25 years, etc), family history of breast cancer in a first-degree relative (yes or no), and body mass index (kg m^−2^, continuous). All tests were two-sided, and *P* values less than 0.05 were considered to be statistically significant.

## Results

### Use of combination oral contraceptive use among control subjects

A total of 73% of 1429 control subjects in these analyses used combination oral contraceptives for a minimum of 6 months prior to interview. Although the proportion of ever-users increased with age (67, 72, and 74% for women aged <35, 35–39, and 40–44 years, respectively), younger women were more likely to have used combination oral contraceptives within 5 years prior to interview (43, 21, 6% for the youngest to oldest age groups).

As shown in Table 2

**Table 2:** Use of combination oral contraceptive (OC) preparations among control subjects

						**Months of use**	
**Oestrogen/progestin content**	**Controls**	**Year of first OC use**	**Age at interview (y)**	**Age at first OC use (y)**	**Any OC**	**Specified OC**	
Any OC use	1086 (100%)	1972	38.5 (4.46)	19.9 (3.21)	68.1 (52.6)	
*Potency*						
High progestin/high oestrogen	130 (12%)	1969	40.6 (2.88)	19.1 (2.54)	72.9 (51.4)	58.5 (41.0)
Low progestin/high oestrogen	270 (25%)	1970	39.7 (3.67)	19.4 (2.65)	74.0 (55.1)	59.3 (45.1)
High progestin/low oestrogen	193 (18%)	1972	39.0 (3.46)	19.8 (3.08)	80.0 (53.5)	62.1 (43.3)
Low progestin/low oestrogen	546 (50%)	1973	36.7 (4.81)	20.0 (3.45)	79.9 (56.8)	67.9 (49.7)
						
*Oestrogen content and dose*						
Mestranol						
>100 *μ*g	212 (20%)	1969	40.6 (3.04)	19.0 (2.60)	72.0 (52.8)	57.6 (42.8)
51–99 *μ*g	141 (13%)	1971	39.1 (3.78)	19.6 (2.79)	75.9 (56.2)	58.3 (40.5)
⩽50 *μ*g	242 (22%)	1973	37.7 (4.05)	19.9 (3.23)	77.2 (52.6)	63.7 (45.2)
Ethinyl oestradiol						
>35 *μ*g	247 (23%)	1972	38.4 (4.29)	20.0 (3.26)	80.1 (55.0)	62.6 (43.9)
⩽35 *μ*g	354 (33%)	1974	35.9 (5.03)	19.8 (3.45)	84.2 (59.2)	67.0 (48.5)
						
*Progestin content*						
Dimethisterone	3 (<1%)	1966	42.3 (1.15)	18.0 (1.73)	75.3 (73.1)	70.2 (78.7)
Chlormadinone acetate	17 (2%)	1966	42.5 (1.62)	19.2 (1.55)	66.9 (65.4)	41.5 (41.5)
Norethynodrel	29 (3%)	1969	40.6 (3.76)	19.0 (3.16)	64.1 (51.6)	45.3 (42.2)
Ethinyl diacetate	180 (17%)	1971	39.6 (3.44)	19.3 (2.83)	73.0 (52.1)	63.9 (48.1)
Norethindrone	663 (61%)	1972	38.0 (4.65)	19.8 (3.23)	72.5 (54.4)	66.1 (50.9)
Norethindrone acetate	70 (6%)	1972	38.2 (3.81)	20.0 (3.24)	81.2 (55.3)	70.7 (48.6)
Norgestrel	247 (23%)	1972	37.6 (4.38)	19.5 (2.93)	83.4 (55.1)	65.4 (46.4)
Levonorgestrel	54 (5%)	1978	33.4 (5.26)	20.8 (4.21)	75.4 (48.6)	55.7 (36.8)

*Note*: With the exception of median year of first oral contraceptive use, this table presents unadjusted means (s.d.) for control subjects who used oral contraceptives for a minimum of 6 months. Oestrogen/progestin categories are not mutually exclusive. For example, a woman who used both mestranol and ethinyl oestradiol preparations is counted in both categories.

, half of control women reported ever using low progestin/low oestrogen potency preparations (50%); however, approximately a third of women took high oestrogen potency (37%, including combination pills with either high or low progestin potency) or high progestin potency pills (30%, including combination pills with either high or low oestrogen potency). Only 12% of women ever used combination oral contraceptives that were of both high progestin and oestrogen potencies. Similar proportions of women took mestranol (55%) and ethinyl oestradiol-containing pills (56%), whereas ever use of preparations containing the progestin norethindrone was reported by the majority of women (61%).

The use of combination oral contraceptive formulations by control subjects in this population paralleled the introduction of new preparations into the US market (Table 2). Women who used either high-potency or high oestrogen dose formulations began pill use earlier in calendar time and were older at interview than those who used lower-potency/dose preparations. For example, the median year at first use of high progestin/high oestrogen potency pills was 1969 with a mean age at diagnosis/interview of 40.6 years, as opposed to first use in 1973 with an average age at diagnosis/interview of 36.7 years for low progestin/low oestrogen formulations. Women who used high-dose or high-potency formulations tended to be slightly younger at the time of first use and used these preparations for a shorter duration, as compared to women taking low-dose or low-potency formulations.

### Hormonal content and potency of combination oral contraceptives and breast cancer risk

Risk of breast cancer associated with combination oral contraceptive use diminished with increasing age (from <35 to 35–44 years) and with time since last use (Table 3

**Table 3:** Combination oral contraceptive use and breast cancer risk

	**<35 years**				**35–44 years**				**Total (<45 years)**			
	**Cases (*N*=267)**	**Controls (*N*=289)**	**RR**	**95% CI**	**Cases (*N* =1373)**	**Controls (*N* =1203)**	**RR**	**95% CI**	**Cases (*N* =1640)**	**Controls (*N* =1492)**	**RR**	**95% CI**
Non users/<6 months of use	58	96	1.00		313	310	1.00		371	406	1.00	
Ever-users	209	193	2.05	1.3–3.2	1060	893	1.12	0.9–1.4	1269	1086	1.24	1.0–1.5
												
*Recency of OC use (relevant to diagnosis/interview)*												
Within 5 years	135	123	2.22	1.4–3.5	174	135	1.30	1.0–1.8	309	258	1.47	1.2–1.9
6–10 years ago	43	39	2.03	1.1–3.7	163	134	1.17	0.9–1.6	206	173	1.33	1.0–1.7
>10 years ago	31	31	1.52	0.8–3.0	723	624	1.07	0.9–1.3	754	655	1.13	0.9–1.4
												
*Content of pill preparation*												
Known	190	184	1.96	1.3–3.0	845	738	1.08	0.9–1.3	1035	922	1.19	1.0–1.4
Unknown	19	9	3.97	1.5–10.6	215	155	1.33	1.0–1.8	234	164	1.50	1.1–1.9

*Note*: RRs and 95% CIs were estimated using maximum likelihood methods and adjusting for age, site, race, menopausal status, a combination variable for age at first birth and number of births, age at menarche, family history of breast cancer, body mass index, and mammography use.

). Since the relationship between oral contraceptive use and breast cancer risk depended on the woman's age at diagnosis (*P*_interaction_<0.05), subsequent analyses are presented within age strata defined by <35 and 35–44 years.

Women younger than 35 years of age who used oral contraceptives within 5 years of interview were at the highest risk of breast cancer with a RR of 2.22. Risk was elevated, although not significantly, among recent oral contraceptive users aged 35–44 years (RR=1.30). Since risk was most elevated among recent oral contraceptive users in this and other populations (Collaborative Group on Hormonal Factors in Breast Cancer, 1996a), we focused our assessment of the effects of dose and potency on pill preparations used for the longest duration in the 5 years prior to interview. Some residual risk existed among women who last used pills 6–10 years prior to interview, particularly among women younger than 35 years; therefore, we also assessed pill preparations used during the 10 years prior to interview and found trends slightly diluted, particularly in the older age group. For brevity, we only present findings among the most recent users (Table 4

**Table 4:** Breast cancer risk associated with hormonal potency and content of combination oral contraceptives used for the longest duration within 5 years of interview

	**<35 years**				**35–44 years**				**Total (<45 years)**			
	**Cases (*N*=193)**	**Controls (*N*=219)**	**RR**	**95% CI**	**Cases (*N*=487)**	**Controls (*N*=445)**	**RR**	**95% CI**	**Cases (*N*=680)**	**Controls (*N*=664)**	**RR**	**95% CI**
Nonusers	58	96	1.00		313	310	1.00		371	406	1.00	
												
*Oestrogen dose*												
Ethinyl estradiol												
⩽35 *μ*g	81	80	1.91	1.1–3.2	80	67	1.02	0.7–1.5	161	147	1.27	0.9–1.7
>35 *μ*g	27	17	3.62	1.7–7.9	32	21	1.52	0.8–2.8	59	38	1.99	1.2–3.2
Mestranol												
⩽50 *μ*g	14	17	1.17	0.5–2.8	29	21	1.52	0.8–2.9	43	38	1.38	0.8–2.3
>50 *μ*g	5	4	2.40	0.5–11.8	12	10	1.25	0.5–3.1	17	14	1.52	0.7–3.3
Unknown	8	5	1.93	0.5–6.8	21	16	1.31	0.6–2.7	29	21	1.52	0.8–2.8
												
*Progestin content*												
Ethynodiol diacetate	12	3	12.0	2.4–59.2	8	10	0.82	0.3–2.2	20	13	1.94	0.9–4.2
Norethindrone	67	73	1.64	1.0–2.8	86	65	1.32	0.9–2.0	153	138	1.35	1.0–1.8
Norethindrone acetate	8	5	3.11	0.9–10.6	17	10	1.77	0.7–4.3	25	15	1.90	0.9–3.8
Norgestrel	21	17	1.97	0.9–4.5	25	23	0.71	0.4–1.3	46	40	1.14	0.7–1.9
Levonorgestrel	20	21	2.12	1.0–4.7	20	12	1.77	0.8–4.0	40	33	1.66	1.0–2.9
Unknown	7	4	2.36	0.6–9.4	18	15	1.30	0.6–2.8	25	19	1.59	0.8–3.1
												
*Progestin potency*												
Low	114	115	1.83	1.1–3.0	140	103	1.28	0.9–1.8	254	218	1.40	1.1–1.8
High	14	4	8.11	2.1–31.6	16	17	0.84	0.4–1.8	30	21	1.54	0.8–2.8
Unknown	7	4	2.35	0.6–9.3	18	15	1.30	0.6–2.8	25	19	1.59	0.8–3.0
												
*Oestrogen potency*												
Low	120	113	1.97	1.2–3.2	138	107	1.18	0.8–1.7	258	220	1.38	1.1–1.8
High	8	6	2.56	0.7–8.9	18	13	1.50	0.7–3.2	26	19	1.70	0.9–3.3
Unknown	7	4	2.32	0.6–9.2	18	15	1.30	0.6–2.8	25	19	1.58	0.8–3.0
												
*Progestin/oestrogen potency*												
Low/low	106	109	1.79	1.1–2.9	126	93	1.27	0.9–1.8	232	202	1.37	1.0–1.8
Low/high	8	6	2.53	0.7–8.8	14	10	1.50	0.6–3.6	22	16	1.71	0.8–3.4
High/low	14	4	8.07	2.1–31.4	12	14	0.71	0.3–1.6	26	18	1.52	0.8–2.9
High/high	0	0	4	3	1.54	0.3–7.3	4	3	1.67	0.4–7.9		
Unknown	7	4	2.34	0.6–9.3	18	15	1.31	0.6–2.8	25	19	1.58	0.8–3.0

*Note*: This table compares nonusers to women who used oral contraceptives within 5 years of interview. Subjects who used oral contraceptives, but not within 5 years of interview, were excluded from analysis (*N*=1788). RRs and 95% CIs were estimated using maximum likelihood methods and adjusting for age, site, race, menopausal status, a combination variable for age at first birth and number of births, age at menarche, family history of breast cancer, body mass index, and mammography use.

).

Among combination oral contraceptive users in this study, younger women were more likely to recall pill brand names: 93% of women younger than 35 years and 81% of those aged 35–44 provided at least one brand of pill that they used. Since breast cancer risk was slightly higher among women who could not recall pill brand names (as shown in Table 3), we included a category for unknown formulation in the final models (Table 4).

Hormonal content and potency of the pill preparation used for the longest duration in the 5 years prior to interview and breast cancer risk is summarised in Table 4. When compared to nonusers, a higher dose of ethinyl oestradiol pills was significantly associated with higher breast cancer risk, overall (*P*_trend_<0.01) and within each age group (*P*_trend_<0.01 for women aged 20–34 years and 0.13 for those aged 35–44 years). Women aged 20–44 years who used pills containing more than 35 *μ*g of ethinyl oestradiol were at an approximately 50% higher risk of breast cancer than recent users of lower-dose preparations when compared to nonusers (respective RRs of 1.99 and 1.27). This relationship was most pronounced in the youngest age group, where risk associated with high- and low-dose ethinyl oestradiol use was 3.62 and 1.91, respectively. Breast cancer risk associated with mestranol was almost entirely explained by doses higher than 50 *μ*g per pill among women younger than 35 years; however, risk was slightly lower among high- compared to low-dose mestranol users in the older age group. With the exception of ethynodiol diacetate (RR=12.0 among women aged 20–34 years based on 12 cases and three controls), risk was similar across all progestin types recently used by women in this study.

In addition to hormonal content, we also examined the relationship between oestrogen and progestin potency and breast cancer risk (Table 4). High progestin potency pills were most strongly related to breast cancer risk among women younger than 35 years of age, with a RR of 8.11 (2.1–31.6). Within this age group, risk was also elevated for recent users of low progestin potency pills (RR=1.83; 95% CI, 1.1–3.0). In the youngest age group, risk of breast cancer increased with higher oestrogen potency pill preparations (*P*_trend_<0.01). Combination progestin/oestrogen potency similarly showed a significant trend of increasing breast cancer risk with increasing potency (*P*_trend_<0.01); recent use of low progestin/low oestrogen potency pills was associated with a RR of 1.79, low progestin/high oestrogen potency with a RR of 2.53, and high progestin/low oestrogen potency with a RR of 8.07. No woman aged younger than 35 years recently used high progestin/high oestrogen potency pills. High progestin/high oestrogen potency pills were used only by women aged 35–44 years (*n*=7), with a RR of 1.54, only slightly higher than the risk associated with low progestin/low oestrogen pills (RR=1.27) in this age group. Similar trends were seen with hormonal potency among women aged 20–44 years (*P*_trend_<0.05), although not as strong as in the youngest age group. The association of breast cancer risk with hormonal content and potency of oral contraceptive preparations was similar to the findings presented in Table 4 for women who used pills for less than 5 years and for those who used them for 5 or more years and after adjusting for frequency of self-breast examination.

## Discussion

In this population-based study of women younger than 45 years of age, we found that breast cancer risk increased with either higher oestrogen dose or higher progestin or oestrogen potency pills used within 5 years of interview, and that these findings were more marked among women younger than 35 years of age. Our findings and those from other studies have shown that recent oral contraceptive use imparts a modest excess in breast cancer risk among women younger than 45 years of age and that this relationship is strongest among very young women (Wingo *et al*, 1993; Collaborative Group on Hormonal Factors in Breast Cancer, 1996a). Previous investigations of oral contraceptive preparations did not integrate these elements (e.g. recency of use and young age at diagnosis) into their study design, but rather included women older than 45 years and assessed lifetime exposure, first used, most used, or last used oral contraceptive preparation, rendering their findings difficult to untangle (Pike *et al*, 1983; Vessey *et al*, 1983,^1989^; Jick *et al*, 1989; UK National Case–Control Study Group, 1989; White *et al*, 1994; Collaborative Group on Hormonal Factors in Breast Cancer, 1996b; Ursin *et al*, 1998; McPherson *et al*, 1987). Nonetheless, the majority of these studies found weak trends, suggesting a higher risk with increasing oestrogen dose, or estrogen/progestin potency among women younger than 45 years of age (Pike *et al*, 1983; McPherson *et al*, 1987; Jick *et al*, 1989; UK National Case–Control Study Group, 1989; White *et al*, 1994; Collaborative Group on Hormonal Factors in Breast Cancer, 1996b; Ursin *et al*, 1998).

Progestins contained in oral contraceptives differ in both dose and potency (Collins, 1994). For example, norethindrone, norethindrone acetate and ethynodiol diacetate are roughly equivalent in potency, while norgestrel is roughly 5–10 times and levonorgestrel is 10–20 times more potent (Dorflinger, 1985). However, progestin doses selected for use in current oral contraceptives are essentially equipotent (Collins, 1994), perhaps explaining the fairly uniform elevation in breast cancer risk across progestin types seen in this study. Since the methods used to determine hormonal potency simultaneously assess the action of both the oestrogen and progestin components, our finding that risk was associated with higher progestin potency, but not specific progestin types, is not contradictory. It is also noteworthy that assigning hormonal potency to different pill formulations is at best an inexact science: potency estimates are derived from a combination of different laboratory methods, none of which are performed regularly in clinical practice (*Drug Facts and Comparisons*, 1997).

In addition to progestational activity, many progestins demonstrate varying degrees of androgenic metabolic effects, being derived from 19 nortestosterone (Mishell, 1989). Levonorgestrel is the most androgenic progestin used in oral contraceptives today followed by norgestrel (Phillips *et al*, 1987); the remaining progestins are essentially devoid of androgen activity (Stubblefield, 1986). Nonetheless, we found no evidence of a trend with increasing androgen activity of pill formulations. Although there was some suggestion that levonorgestrel preparations imparted one of the highest risks (RR=1.77) among women aged 35–44 years, this finding was not evident among younger study participants.

One of the weaknesses of this study was that pill brand name was based on subject recall. Validation studies have shown that recall of oral contraceptive use and timeframe of use is accurate, but that recall of specific brand names is less accurate (Coulter *et al*, 1986; Harlow and Linet, 1989). By employing structured, in-person interviews using a combination of family planning calendars to aid recall and comprehensive picture books of oral contraceptive preparations, a fairly high percentage of oral contraceptive users (83%) provided information on pill brand names, and 93% of those younger than 35 years. Since slightly more cases (18%) as compared to controls (15%) using oral contraceptives provided information on pill brand names, and risk of breast cancer among those with unknown formulations was also slightly higher, we adjusted for subjects with unknown formulations in our analysis.

Since their inception, oral contraceptive pills have undergone substantial changes in formulation. For example, the FDA reported that in 1964, 94% of the oral contraceptives dispensed by retail pharmacies were high oestrogen potency pills, and by 1984, 85% of oral contraceptives dispensed were low oestrogen potency pills (Piper and Kennedy, 1987); the oestrogen in the older formulations contained mestranol, while all those developed since 1974 contain ethinyl oestradiol (Mishell, 1989; Wilson *et al*, 1998). Analysis of the use of combination oral contraceptives among control subjects parallel the introduction of new pill formulations into the US market, suggesting that the quality of the pill brand names recalled may be quite accurate. Among control subjects who used oral contraceptives, the median year of first use of oral contraceptives for women who used high oestrogen potency, high dose, or mestranol-containing pills was earlier than for users of low potency, low dose, or ethinyl oestradiol pills. Similarly, progestins removed from the market prior to 1980, including chlormadinone acetate, dimethisterone, and medroxyprogesterone acetate, and norethynodrel, which was no longer marketed as of 1988, were used by women who first took the pill earlier in calendar time than those using norgestrel and levonorgestrel-containing pills that are available in current formulations (Piper and Kennedy, 1987).

Although it is possible that the modest increase in breast cancer risk associated with recent use of higher oestrogen dose or high- potency pills is explainable by a detection bias, several plausible biologic mechanisms exist. The steroid hormones of oral contraceptives, oestrogen and 19-nortestosterone-derived progestogens, have long been shown to stimulate breast cell mitotic activity by acting directly, alone or synergistically, on breast cells *in vivo* (Jeng *et al*, 1992; Isaksson *et al*, 2001). The collaborative reanalysis examined the use of screening mammography among controls and found no evidence that recent users of oral contraceptives were more likely to report having had a mammogram than never-users (Collaborative Group on Hormonal Factors in Breast Cancer, 1996b). In addition, our findings do not appear to be susceptible to a surveillance bias, as the estimates were similar after adjustment for number of mammograms and for frequency of self-breast examinations in the 5 years prior to diagnosis.

Although our findings suggest that newer low oestrogen dose/low-potency oral contraceptives may impart a lower risk of breast cancer than that associated with earlier high-dose/high-potency preparations, we only had small numbers of women within strata of specific pill preparations. Thus, reanalysis of earlier studies (Pike *et al*, 1983; Vessey *et al*, 1983,^1989^; McPherson *et al*, 1987; Jick *et al*, 1989; UK National Case–Control Study Group, 1989; White *et al*, 1994; Collaborative Group on Hormonal Factors in Breast Cancer, 1996b; Ursin *et al*, 1998;) or analysis of formulation data from earlier studies that has not yet been published (Meirik *et al*, 1986; Paul *et al*, 1990; Weinstein *et al*, 1991; Wingo *et al*, 1993; Primic-Zakelj *et al*, 1995) would help to confirm our findings.

Our analysis examines formulations of oral contraceptive pills used prior to 1993. Since that time new major developments in pill preparations include widespread use of third-generation progestins with low androgenic activity, and further reduction in the dosage of ethinyl oestradiol from 35 to 20 *μ*g per pill (Burkman, 2001). Since oral contraceptives are used by 80% of women at one point during their reproductive lives, and women are now using the pill for longer than ever before (Piccinino and Mosher, 1998), even modest increases in risks associated with use can have a large public health impact. New studies are necessary to assess whether there is risk associated with newer oral contraceptive pill formulations, including progestin-only preparations, which have claimed an increasing market in recent times.
